# Association between work sick-leave absenteeism and SARS-CoV-2 notifications in the Netherlands during the COVID-19 epidemic

**DOI:** 10.1093/eurpub/ckae051

**Published:** 2024-03-21

**Authors:** Martijn G Keet, Bronke Boudewijns, Femke Jongenotter, Senna van Iersel, Cornelis H van Werkhoven, Rianne B van Gageldonk-Lafeber, Bram W Wisse, Liselotte van Asten

**Affiliations:** Centre for Infectious Disease Control Netherlands, National Institute for Public Health and the Environment (RIVM), Bilthoven, The Netherlands; Centre for Infectious Disease Control Netherlands, National Institute for Public Health and the Environment (RIVM), Bilthoven, The Netherlands; Centre for Infectious Disease Control Netherlands, National Institute for Public Health and the Environment (RIVM), Bilthoven, The Netherlands; Centre for Infectious Disease Control Netherlands, National Institute for Public Health and the Environment (RIVM), Bilthoven, The Netherlands; Centre for Infectious Disease Control Netherlands, National Institute for Public Health and the Environment (RIVM), Bilthoven, The Netherlands; Julius Center for Health Sciences and Primary Care, University Medical Center Utrecht, Utrecht University, Utrecht, The Netherlands; Centre for Infectious Disease Control Netherlands, National Institute for Public Health and the Environment (RIVM), Bilthoven, The Netherlands; Research and Business Development, HumanTotalCare (HTC), Utrecht, The Netherlands; Centre for Infectious Disease Control Netherlands, National Institute for Public Health and the Environment (RIVM), Bilthoven, The Netherlands

## Abstract

**Background:**

Alternative data sources for surveillance have gained importance in maintaining coronavirus disease 2019 (COVID-19) situational awareness as nationwide testing has drastically decreased. Therefore, we explored whether rates of sick-leave from work are associated with severe acute respiratory syndrome coronavirus 2 (SARS-CoV-2) notification trends and at which lag, to indicate the usefulness of sick-leave data for COVID-19 surveillance.

**Methods:**

We explored trends during the COVID-19 epidemic of weekly sick-leave rates and SARS-CoV-2 notification rates from 1 June 2020 to 10 April 2022. Separate time series were inspected visually. Then, Spearman correlation coefficients were calculated at different lag and lead times of zero to four weeks between sick-leave and SARS-CoV-2 notification rates. We distinguished between four SARS-CoV-2 variant periods, two labour sectors and overall, and all-cause sick-leave versus COVID-19-specific sick-leave.

**Results:**

The correlation coefficients between weekly all-cause sick-leave and SARS-CoV-2 notification rate at optimal lags were between 0.58 and 0.93, varying by the variant period and sector (overall: 0.83, lag −1; 95% CI [0.76, 0.88]). COVID-19-specific sick-leave correlations were higher than all-cause sick-leave correlations. Correlations were slightly lower in healthcare and education than overall. The highest correlations were mostly at lag −2 and −1 for all-cause sick-leave, meaning that sick-leave preceded SARS-CoV-2 notifications. Correlations were highest mostly at lag zero for COVID-19-specific sick-leave (coinciding with SARS-CoV-2 notifications).

**Conclusion:**

All-cause sick-leave might offer an earlier indication and evolution of trends in SARS-CoV-2 rates, especially when testing is less available. Sick-leave data may complement COVID-19 and other infectious disease surveillance systems as a syndromic data source.

## Introduction

Extensive monitoring of severe acute respiratory syndrome coronavirus 2 (SARS-CoV-2) activity was in place in the Netherlands since the first infection was detected on 27 February 2020. This monitoring system included traditional epidemiological counts such as SARS-CoV-2 positive laboratory diagnoses, hospitalizations, ICU admissions and less traditional markers such as virus particle concentration in sewage water and community self-reported symptoms by web application (‘Infectieradar’).^1^^,^^2^ Large scale testing was mostly done at Public Health Services (PHS) coronavirus disease 2019 (COVID-19) test facilities free of charge, in persons with symptoms (as of 1 June 2020), those who had been in contact with a SARS-CoV-2 positive person irrespective of symptoms (as of 1 December 2020), and after a positive self-test (as of 3 February 2021). Over time the willingness to test at an official testing location decreased,^3^ which reduces the value of the data for surveillance and likely biases it towards certain risk groups. Since 10 April 2022, confirmation of a positive self-test was no longer required and infection notifications with detailed additional information were replaced by notifications with basic information (sex, date of birth, zip code and date of test result). Due to the reduction in information per notification and the termination of mandatory reporting per 1 July 2023, non-traditional surveillance methods will become more prominent as a marker for spread of mild disease in the general population.

A possibly viable data source is work absenteeism, specifically sick-leave. Advantages of using sick-leave data for surveillance could be timeliness, availability, and versatility to use it for monitoring of many infectious diseases. COVID-19 surveillance was relatively timely based on detected infections in the community. Given that the notification of SARS-CoV-2 infections is no longer mandatory it might take longer to detect changes in SARS-CoV-2 activity, through the monitoring of for example hospitalizations. Hospitalizations represent severe cases and are monitored, but are not mandatory notifiable. Such hospital admissions lag behind the symptom onset.^4^ Sick-leave data are collected for business purposes, and this registration will be maintained even when SARS-CoV-2 testing decreases. The COVID-19 pandemic likely has impacted sick-leave among the working population worldwide.^5–8^ If sick-leave is associated with SARS-CoV-2, then it is to be expected that it could also be associated with other infectious diseases such as influenza, as a few studies suggest.^9–14^

Sick-leave data also provide not previously used information in infectious disease surveillance in the Netherlands. In addition to its potential to reflect disease trends during an epidemic and to increase situational awareness, sick-leave trends could be used to estimate disease trends at the start of an epidemic, when laboratory testing for a new pathogen is not yet available.

To date, few studies have been published on the use of sick-leave data as a surveillance tool for infectious diseases. To understand the trend and timing of sick-leave relative to SARS-CoV-2 trends in the Netherlands we compared weekly sick-leave to SARS-CoV-2 notification rates during the COVID-19 epidemic.

## Methods

### Design and setting

For this descriptive, ecological analysis, we used sick-leave data and notifications of laboratory-confirmed SARS-CoV-2 infections from the national COVID-19 surveillance database in the period of 1 June 2020 to 10 April 2022. The first months of the COVID-19 epidemic, March-May 2020, were excluded as laboratory testing capacity was limited.

### Sick-leave data

Anonymized sick-leave data were made available by Human Total Care (HTC), a nationwide Dutch occupational health service.^15^ HTC provides guidance and support to both employees and their employers during sick-leave and in the return to work. For these services, the employers report the sick-leaves of their employees to HTC. HTC does not receive data from their contracted employers on non-illness absenteeism of employees, such as holidays or care leave. Depending on the employer contract, some employees receive an additional triage questionnaire at the start of the sick-leave. In this questionnaire the reason for sick-leave is reported by the employee (Supplementary file S1). During the COVID-19 epidemic additional COVID-19 triage questions were added to this questionnaire. HTC covers approximately 11% of the Dutch working population as defined by Statistics Netherlands.^15^

We used the date of first day of sick-leave (without illness duration), work sector and absence cause. We categorized healthcare and education employees according to the Dutch Standard Industrial Classification.^16^ Healthcare was of special interest due to the additional pressure of sick-leave on the sector and education because of the societal impact of class and school closures that would result from sick-leave. All-cause sick-leave (AC-sick-leave) was defined as all sick-leave, while COVID-19-specific sick-leave (CS-sick-leave) was defined as reports with a direct or indirect laboratory-confirmed SARS-CoV-2 infection as the sick-leave reason (questionnaire subset). Weekly sick-leave rates per 100 000 persons were calculated by dividing counts by the coverage data (i.e. the total number of employees of the employers contracting HTC services).

### COVID-19 surveillance data

SARS-CoV-2 notifications were defined as laboratory-confirmed positive SARS-CoV-2 tests. Tests were performed by (non-)commercial test facilities or healthcare facilities. Mandatory reporting of these infections was done to the regional PHS in the Netherlands and reported via OSIRIS (Online System for Infectious disease Reporting) to the National Institute of Public Health and the Environment (RIVM).^1^

We based SARS-CoV-2 notification on the date of positive test result and selected aged 18–66 to best match the working population, as 66 is the retirement age in the Netherlands.^17^ Persons reporting unemployment were excluded. Reports with unknown employment were included as they constitute a mix of employed and unemployed persons with missing data (less detailed employment registrations with time; Supplementary file S2) and groups such as students. Because of the before-mentioned societal interest in healthcare and education sectors, SARS-CoV-2 infected people were specifically asked whether they were employed in either profession. Consequently, availability of employment data was high for these sectors in OSIRIS. Persons working in ‘(health)care in hospital’, ‘(health)care in nursing home/residential care facility for the elderly’, ‘(health)care in other institution with 24-hour care’, ‘home (health)care’ or ‘other (health)care’ were classified as healthcare workers. Those working in ‘day-care’, ‘primary school or after-school care’, ‘secondary education’, ‘vocational college’ and ‘higher education’ were classified as education workers. Weekly SARS-CoV-2 notification rates per 100 000 persons were calculated using quarterly labour force size data from Statistics Netherlands.^18^

### Data exploration

Data were explored using descriptive statistics and visual inspection of time series graphs; overall and separately for healthcare and education sectors.

To derive plausible correlations during the Christmas holidays in these retrospective analyses, due to potentially different lags per period, we replaced the observed sick-leave rates during Christmas holidays with the mean of the sick-leave rates in the one to two weeks prior to and after the holiday for all categories of sick-leave (as not to dilute correlations). Employees that are on holidays are less likely to report sick-leave, but are still fully counted in the coverage data (personal communication HTC). This type of interpolation was also previously done by others.^19^ Additionally, for education, this was also done for all other official one- or two-week school holidays.^20^ This was not done for the six-week summer holiday, as we assumed these interpolations less reliable for longer time periods. For one-week holidays, we interpolated the sick-leave rates as the mean sick-leave rates of the week before and after the holiday week. For two-week holidays, the first week was interpolated using the mean of the two weeks before and one week after the holiday; the second week was interpolated using the mean of one week before and two weeks after the holiday. The middle week of the extended three-week long Christmas holiday in 2021 was interpolated using the mean of the single week directly before and after the holiday.

### Statistical analysis

We calculated the Spearman’s rank correlation coefficients between the weekly sick-leave by first date of absence and SARS-CoV-2 notification rates by date of positive test result. To gain insight into the timing of the two time series relative to each other we also calculated the correlation coefficients at different time lags (zero to four weeks). Positive lags indicate that sick-leave occurred after SARS-CoV-2 notifications, while negative lags indicate that sick-leave preceded SARS-CoV-2 notifications. We categorized correlation correlations by four time periods based on dominant SARS-CoV-2 variants, reflecting variances in infectiousness, disease severity and policy changes. These periods were defined compliant with periods previously defined by the RIVM, which considered variant dominance, hospital admissions and evolving factors like policy changes and vaccination rates.^21^

- Wildtype: 01 June 2020 until 31 June 2021
- *Alpha*: 01 February 2021 until 04 July 2021
- *Delta*: 05 July 2021 until 02 January 2022
- *Omicron*: 03 January 2022 until 10 April 2022

## Results

### General characteristics

In total 5 738 139 SARS-CoV-2 infections were notified in the Netherlands during the study period (table 1). For 2 177 219 notifications (37.9%) the labour sector was known, of which 289 674 (13.3%) worked in healthcare and 138 869 (6.4%) in education.

**Table 1:** Total number of registered sick-leave and SARS-CoV-2 notifications (aged 18–66) per labour sector during the study period^a^

Labour sector	SARS-CoV-2 dataset	Sick-leave dataset			
	Notifications	COVID-19-specific	Other cause	Cause unknown^b^	Total
Healthcare, *n* (%)^c^	289 674 (13.3%)	6601 (16.6%)	11 882 (11.5%)	117 502 (11.4%)	135 985 (11.6%)
Education, *n* (%)^c^	138 869 (6.4%)	908 (2.3%)	1854 (1.8%)	80 709 (7.9%)	83 471 (7.1%)
Other, *n* (%)^c^	1 748 876 (80.3%)	32 254 (81.1%)	89 591 (86.7%)	828 863 (80.7%)	950 708 (81.2%)
Unknown, *n* (%)	3 560 920 (62.1%)	1232 (3.0%)	2213 (2.1%)	62 267 (5.7%)	65 712 (5.3%)
Total	5 738 139	40 995 (3.3%)	105 540 (8.5%)	1 089 341 (88.1%)	1 235 876

a From separate work sick-leave dataset and SARS-CoV-2 infection notification dataset (not linkable at individual level).

b Contains both cases registered with unknown cause and cases where the cause was not registered.

c % based on totals excluding unknown.

In total, 1 235 876 sick-leave reports were registered, of which 146 535 (11.9%) included a sick-leave reason (table 1). CS-sick-leave was reported for 40 995 sick-leave reports (28.0% of reports with a sick-leave reason, 3.3% of all reports), of which 28 637 (69.9%) were based on a positive SARS-CoV-2 test and the remainder was reported as suspected SARS-CoV-2 infection. For 1 170 164 (94.7%) reports the labour sector was known: 135 985 (11.6%) worked in healthcare and 83 471 (7.1%) in education.

The average weekly AC-sick-leave rate was higher than SARS-CoV-2 weekly notification rate during all periods except the *omicron* period (Supplementary file S3).

The age distribution of sick-leave reports had two peaks around ages 30 and 50, while the age distribution of the SARS-CoV-2 notifications showed a higher proportion of young people (Supplementary files S4-5).

### Overall time series and correlations

**Figure 1:**
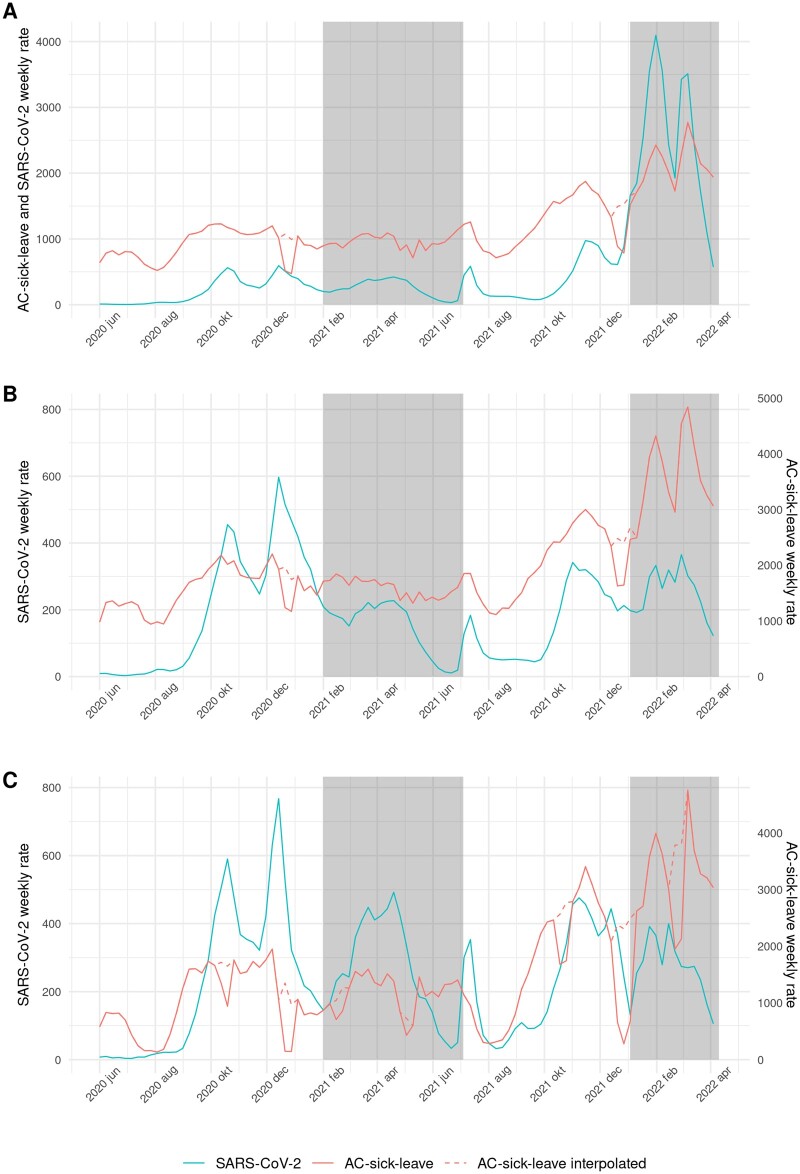
All-cause (AC-)sick-leave and SARS-CoV-2 weekly rates per 100 000. Red dashed line: AC-sickleave is interpolated during Christmas holidays (all short holidays in the education sector). White and grey shading: the periods of SARS-CoV-2 variants are shown with alternating shaded planes (wildtype, alpha, delta, omicron). A. Overall, B. Healthcare sector and C. Education sector

Visual inspection of the overall time series (all work sectors combined) showed some coherence between AC-sick-leave and SARS-CoV-2 weekly rates, seemingly more pronounced during the *delta* and *omicron* periods (figure 1A). Changes in AC-sick-leave and SARS-CoV-2 rates were often concurrent: the first two SARS-CoV-2 peaks in 2020 roughly coincided with increases in AC-sick-leave and the four SARS-CoV-2 peaks during the *delta* and *omicron* periods roughly coincided with more pronounced peaks in AC-sick-leave. In August 2020 and in August/September 2021, AC-sick-leave increased before the SARS-CoV-2 rate did. This is also reflected by the overall correlation being the highest (0.83, 95% CI [0.76, 0.88]) at lag −1, which means that AC-sick-leave preceded SARS-CoV-2 notifications by one week (table 2). Correlation coefficient size and optimal lag varied by variant period. The wildtype (0.87, 95% CI [0.76, 0.93]) and *delta* (0.82, 95% CI [0.63, 0.92]) periods showed higher correlations than *alpha* (0.72, 95% CI [0.42, 0.87]) and *omicron* (0.66, 95% CI [0.19, 0.88]). Optimal lags were negative for all periods except the *omicron* period (+1).

**Table 2:** Highest Spearman correlation coefficients between sick-leave and SARS-CoV-2 weekly rate per 100 000 at optimal lags^a^ during the study period

Sector		Overall		Healthcare		Education	
Type of sick-leave		All-cause	COVID-19-specific	All-cause	COVID-19-specific	All-cause	COVID-19-specific
Total study period	Optimal lag	−1	0	−1	0	−2	0
	Correlation coefficient	0.83, 95% CI [0.76, 0.88]	0.97, 95% CI [0.95, 0.98]	0.71, 95% CI [0.59, 0.79]	0.80, 95% CI [0.72, 0.86]	0.61, 95% CI [0.47, 0.72]	0.70, 95% CI [0.59, 0.80]
Wild-type period	Optimal lag	−2	0	−2	0	−2	0
	Correlation coefficient	0.87, 95% CI [0.75, 0.93]	0.95, 95% CI [0.78, 0.94]	0.88, 95% CI [0.77, 0.94]	0.90, 95% CI [0.82, 0.95]	0.85, 95% CI [0.72, 0.92]	0.79, 95% CI [0.62, 0.89]
Alpha period	Optimal lag	−2	0	−4	0	−2	0
	Correlation coefficient	0.72, 95% CI [0.42, 0.87]	0.97, 95% CI [0.92, 0.99]	0.76, 95% CI [0.50, 0.90]	0.81, 95% CI [0.59, 0.92]	0.58, 95% CI [0.20, 0.80]	0.75, 95% CI [0.47, 0.89]
Delta period	Optimal lag	−2	0	−1	0	−2	0
	Correlation coefficient	0.81, 95% CI [0.59, 0.92]	0.96, 95% CI [0.91, 0.98]	0.89, 95% CI [0.76, 0.95]	0.87, 95% CI [0.73, 0.94]	0.93, 95% CI [0.84, 0.97]	0.88, 95% CI [0.74, 0.94]
Omicron period	Optimal lag	+1	0	0	0	+1	+1
	Correlation coefficient	0.66, 95% CI [0.19, 0.88]	0.66, 95% CI [0.19, 0.88]	0.73, 95% CI [0.33, 0.91]	0.76, 95% CI [0.39, 0.92]	0.68, 95% CI [0.23, 0.89]	0.64, 95% CI [0.16, 0.87]

a Lag in weeks; the optimal lag being the lag with the highest correlation coefficient (negative lags: sick-leave in the weeks preceding SARS-CoV-2 weekly notification rate, positive lags: sick-leave in the weeks after SARS-CoV-2 weekly notification rate).

**Figure 2:**
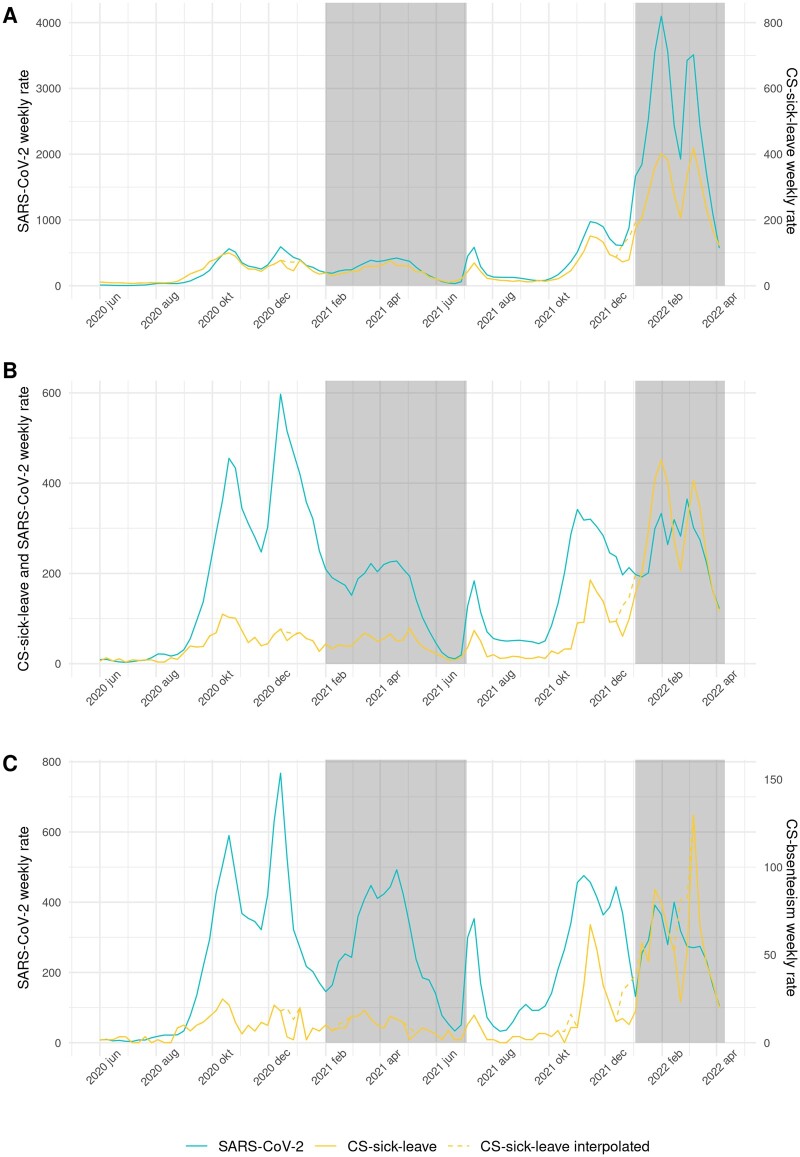
COVID-specific (CS-)sick-leave and SARS-CoV-2 weekly rates per 100 000. Yellow dashed line: CS-sick-leave is interpolated during Christmas holidays (all short holidays in the education sector). White and grey shading: the periods of SARS-CoV-2 variants are shown with alternating shaded planes (wildtype, alpha, delta, omicron). A. Overall, B. Healthcare sector and C. Education sector

Overall CS-sick-leave rates followed an almost identical trend to the SARS-CoV-2 rate (figure 2A). In August 2020, the rise in CS-sick-leave visually preceded the rise in SARS-CoV-2 rates. During the rest of the study period, the time series seemingly coincided, as reflected by the correlations being highest at lag zero. This indicates no delay between CS-sick-leave and SARS-CoV-2 rates. CS-sick-leave showed higher correlation with the SARS-CoV-2 rate than AC-sick-leave in all periods (table 2). While CS-sick-leave peaks were very pronounced during the *omicron* period, the SARS-CoV-2 rate was even more pronounced.

### Healthcare

The time series for the healthcare sector resembled the overall time series (figure 1B) at the same optimal lag of −1 but with lower correlations (0.71, 95% CI [0.59, 0.79], table 2).

The correlations between the CS-sick-leave and SARS-CoV-2 rates were similar to the correlations between AC-sick-leave and SARS-CoV-2 (figure 2B), with a higher correlation for all periods except *delta*. The optimal lags were zero (table 2).

### Education

For the education sector, AC-sick-leave and SARS-CoV-2 weekly rates trends coincided for shorter durations and not consistently over the study period (figure 1C). This was also reflected by the lower correlation (0.61, 95% CI [0.47, 0.72]) than overall and in healthcare. The optimal lag was −2 for the total study period (table 2).

The trend and peaks of weekly CS-sick-leave and SARS-CoV-2 rates were more similar to each other during all periods than AC-sick-leave was (figure 2C). The optimal lags were either zero or +1 (table 2).

## Discussion

This study shows an association between weekly sick-leave and SARS-CoV-2 infection notification rates and thus gives an indication that sick-leave data are potentially a worthwhile additional data source for strengthening COVID-19 surveillance. This association also indicates that sick-leave data may be useful as an additional surveillance source for other respiratory infectious diseases as well as for pandemic preparedness.

Trends between sick-leave and SARS-CoV-2 notification rates were relatively similar and the correlations between the two were moderate to high, varying by SARS-CoV-2 variant.

AC-sick-leave, while showing a lower association with SARS-CoV-2 than CS-sick-leave, seems a more timely indicator of ensuing SARS-CoV-2 increases. AC-sick-leave data are also more widely available than CS-sick-leave data. Data from healthcare and education sectors did not provide increased timeliness nor stronger correlations.

In all periods except *omicron*, changes in AC-sick-leave preceded changes in SARS-CoV-2 notifications as judged from the optimal lag of the correlations. Sick-leave reports, which can be registered before individuals get tested or before waiting to receive test results, are likely to precede SARS-CoV-2 notifications. This result is in agreement with the findings by Gómez *et al.* that showed sick-leave among nursing home employees was more timely than COVID-19 cases.^7^

Our results suggest that the less specific AC-sick-leave may be timelier than the more specific CS-sick-leave. One explanation is that at the start of a wave, less employees attribute their illness to COVID-19 and therefore do not get tested. This explanation is supported by behavioural research which indicates that per wave, the willingness to test increased as more SARS-CoV-2 infections were identified within the wave.^22^

An explanation for CS-sick-leave showing a higher correlation with SARS-CoV-2 notifications is that the data are highly linked: 69.9% of CS-sick-leave was registered as due to a positive SARS-CoV-2 test. Additionally, for the remaining group suspecting COVID-19, this was based on a close contact’s positive test or awaiting their test result.

While disease specific sick-leave provides more precise information on disease spread, this requires laboratory testing, complicating the consistent acquisition of this information. CS-sick-leave data were less abundant than AC-sick-leave data: the sick-leave symptom or cause was available for only 11.9% of the study population, and 28% of that subset reported suspected or confirmed SARS-CoV-2. More importantly, the COVID-19 questionnaire has been discontinued since 01 April 2023.

With few reports on the potential timeliness of work sick-leave data for infectious disease surveillance, our study is the first to explore the lag time between the weekly sick-leave and SARS-CoV-2 rates. However, while we showed the results at the optimal lags, nearby lags often showed similar correlation coefficients. Therefore, the optimal lag only provides an indication of the timeliness of one time series relative to another.

Our results suggest that the degree of association between sick-leave and SARS-CoV-2 trends differs between SARS-CoV-2 variants. This disparity could be due to differences in virus characteristics, vaccine uptake and natural immunity in the population. The ratio of average CS-sick-leave to SARS-CoV-2 notification rates was lower for every new variant in the entire study population, especially during the *omicron* period. This points to a reduced infection severity and is in accordance with studies showing that the *omicron* variant is less severe and includes more asymptomatic infections.^23–25^ Vaccine coverage and naturally acquired population immunity also impacts the difference between periods. It has been shown that vaccination for SARS-CoV-2 has led to milder infections and less sick-leave in healthcare personnel.^26^^,^^27^ Vaccine coverage was mainly a factor in the *delta* and *omicron* periods and this may have impacted the correlation between sick-leave and SARS-CoV-2 rates. For example, a slight increase in sick-leaves due to asymptomatic confirmed SARS-CoV-2 infections was found in fully vaccinated healthcare personnel compared to partially/non-vaccinated personnel.^27^ As asymptomatic infections are harder to detect, vaccinated employees may provide less insight into the spread of SARS-CoV-2. Furthermore, asymptomatic infections will not cause any sick-leave if testing possibilities are not present as at the start of an epidemic or not used as in an endemic phase.

Differences in the associations between variants may also have been influenced by calendar time. Sick-leave usually rises in the run-up to the respiratory infection season, increasing the correlation coefficients if the SARS-CoV-2 rate coincided with the respiratory season. Specifically in the winter of 2021/2022, an influenza epidemic occurred in the Netherlands,^28^ partially overlapping with the *omicron* wave and potentially watering down the correlation between sick-leave and SARS-CoV-2 rates. Other conditions, such as seasonal affective disorder cause more sick-leave during the winter, thus also coinciding with the respiratory season in the Netherlands.^29^ Such time-dependent phenomena can influence the size of the estimated correlations for all periods.

While the interpolation during holidays is used to estimate a plausible correlation unaffected by holidays, the estimated correlation is not valid during holidays.

One important limitation of the data is that sick-leave might be influenced by the changing control measures imposed by the government, especially the advice to work from home (WFH). This advice was active in some form from before the start of the study period until 15 March 2022.^30^ Employees able to WFH might be more likely to keep on working whilst having COVID-19 symptoms when they would otherwise have called in sick. Healthcare workers were much less influenced by this control measure, and education employees were dependent on school closures.

We were not able to incorporate positive self-test results. On 31 March 2021 self-tests were encouraged to be used as a substitute first test by persons without symptoms. From 03 December 2021 self-tests were encouraged as a first test in all situations.^31^ Although people with a positive self-test were advised to get laboratory confirmation, it can be assumed that many did not and thus the actual weekly SARS-CoV-2 rate was likely higher than reported.

Finally, the correlations by labour force are less representative for the total population during the *omicron* period, as the registration of workplace data in the COVID-19 surveillance was greatly reduced (Supplementary file S2). This led to an underestimation of the SARS-CoV-2 rate in the education and healthcare sectors during this period.

As our analyses were based on the first day of absence and day of positive test result. For both variables a reporting delay is to be expected in a prospective surveillance setting. The sick-leave registration delay is three-fold. First, reporting in sick to your employer might take time from the date of symptom onset, although this delay was likely to be short during the COVID-19 epidemic. Second, there can be a delay between calling in sick at the employer and registration at HTC. Nearly half of all absence reports were registered on the same day and 36% were registered within a week (constant over time; Supplementary file S6). Thirdly, reporting from HTC to RIVM is expected to cost an additional day.

SARS-CoV-2 notifications are firstly delayed due to the time period between infection suspicion and actual testing at a test location. One study found this delay to be two days or more for over half of the participants.^32^ When testing capacity became insufficient during high incidence phases of the epidemic (November 2021, January 2022), it could take up to two days for someone to get a test appointment. Further delay can occur for receiving the positive test result; generally one to two days.^33^ The final delay is in the OSIRIS updating time, at 10:00 every morning. Thus most are registered one day after test result availability (Supplementary file S7).

Spearman correlation coefficients and lags when including registration delay of sick-leave and publication delay of SARS-CoV-2 notifications were very similar to the presented results (Supplementary file S8).

Few studies on the use of work sick-leave data for infectious disease surveillance have been reported. Due to the descriptive nature of this study further research is warranted on the use of sick-leave data for infectious disease surveillance and pandemic preparedness. We expect such data to increase general situational awareness, potentially be of use as early warning for common circulating pathogens and be available to be used before testing to an emerging pathogen is possible. An additional benefit is potentially timelier and more thorough monitoring of pressure on the healthcare system during an epidemic, as personnel availability plays a large role during epidemics.

In the COVID-19 surveillance, signals and trends are compared across multiple sources. The addition of sick-leave data to this surveillance offers opportunities for strengthening this surveillance and infectious disease surveillance in general. During the majority of this study period there was little to no circulation of other respiratory viruses, such as influenza.^34^ If there are multiple pathogens circulating, then sick-leave rates will not reflect a single pathogen’s trends. As sick-leave data are syndromic and thus non-specific to COVID-19, a similar association might be expected with other respiratory infections during epidemic phases when one pathogen is dominantly circulating.

## Conclusion

This study shows that work sick-leave rates were associated with SARS-CoV-2 notification rates and thus an indicator of SARS-CoV-2 activity. Additionally, sick-leave is potentially two weeks timelier than laboratory surveillance. Sick-leave may be a useful, and to date underutilized, data source for infectious disease surveillance. We expect the value of sick-leave can be increased by an improved documentation of sick-leave reasons. Sick-leave data may improve situational awareness in future pandemics, specifically when laboratory testing is not yet possible.

## Supplementary Material

ckae051_Supplementary_Data

## Data Availability

The sick-leave dataset underlying this study are not publicly available as they are owned by a third party (HumanTotalCare, HTC). On reasonable request the dataset used for this study can be requested from HTC. The metadata (except occupation data) of the SARS-CoV-2 dataset underlying this study are publicly available in RIVMdata at data.rivm.nl/covid-19.
