# Supplementary material for: Association between work sick-leave absenteeism and SARS-CoV-2 notifications in the Netherlands during the COVID-19 epidemic
Source: Eur J Public Health. 2024 Mar 21;34(3):497–504. doi: 10.1093/eurpub/ckae051 (PMC11161148; doi:10.1093/eurpub/ckae051)
Supplement: ckae051_Supplementary_Data [file ckae051_supplementary_data.zip › ckae051_Supplementary_Data/ejph-2023-08-om-0463-File004.pdf]

Supplementary file S1. Categories of sick-leave registered by a subset of employers contracting HTC for occupational health services (covering 12% of employees in the study).

- Corona (including suspicion)[a]
- Influenza or flu-like complaints
- Physical health complaints (for example: injuries, cardiovascular disease or complaints to airways or abdomen)
- Mental health and/or stress-related health complaints
- Complaints related to pregnancy or childbirth
- Awaiting an operation
- Recovering from an operation
- Other health complaints

a Infected with SARS-CoV-2 or sick with COVID-19 (including confirmed and suspicion)
