# Supplementary material for: Association between work sick-leave absenteeism and SARS-CoV-2 notifications in the Netherlands during the COVID-19 epidemic
Source: Eur J Public Health. 2024 Mar 21;34(3):497–504. doi: 10.1093/eurpub/ckae051 (PMC11161148; doi:10.1093/eurpub/ckae051)
Supplement: ckae051_Supplementary_Data [file ckae051_supplementary_data.zip › ckae051_Supplementary_Data/ejph-2023-08-om-0463-File005.pdf]

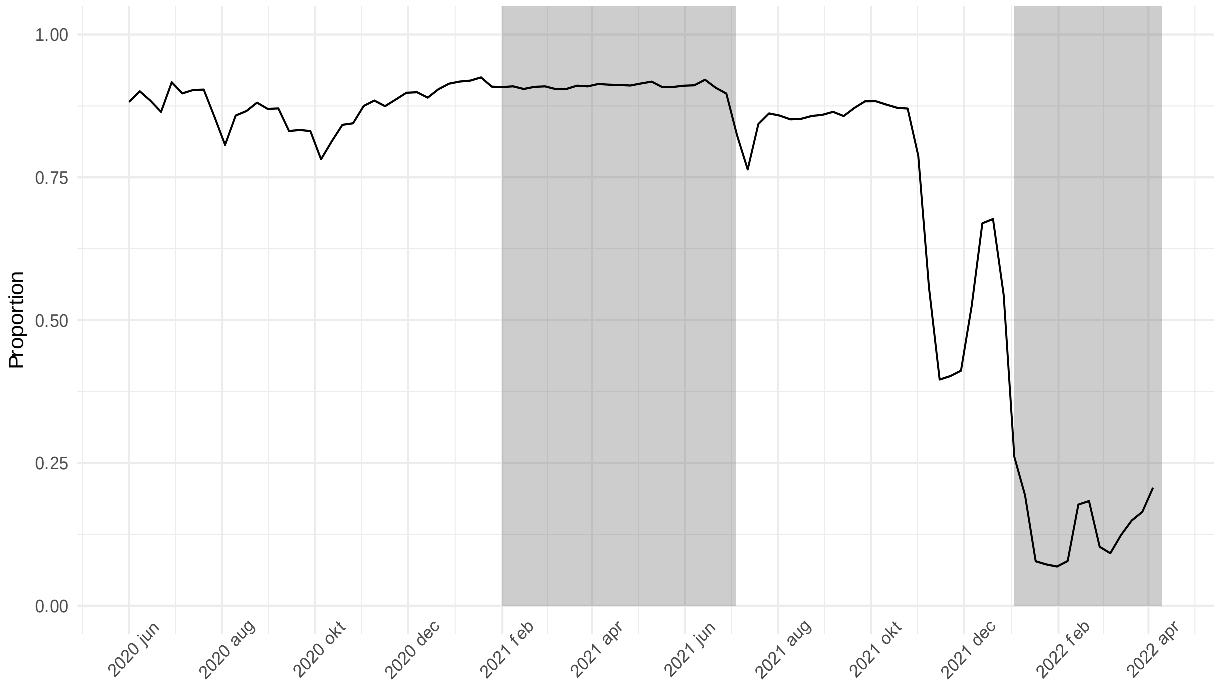

Supplementary file S2. Proportion of SARS-CoV-2 notifications with known workplace information over time. *The average coverage per period is: 69% (total), 88% (wildtype), 91% (alpha), 68% (delta) and 14% (omicron; weighted by total notifications, not an average of all weeks).*
