# Supplementary material for: Association between work sick-leave absenteeism and SARS-CoV-2 notifications in the Netherlands during the COVID-19 epidemic
Source: Eur J Public Health. 2024 Mar 21;34(3):497–504. doi: 10.1093/eurpub/ckae051 (PMC11161148; doi:10.1093/eurpub/ckae051)
Supplement: ckae051_Supplementary_Data [file ckae051_supplementary_data.zip › ckae051_Supplementary_Data/ejph-2023-08-om-0463-File006.pdf]

Supplementary file S3. Average weekly rate per 100,000 of sick-leave and SARS-CoV-2 notifications shown per labour sector and period.

		Average weekly rate per 100,000				
**Labour sector**	**Dataset**	**Total study period**	**Wildtype period**	***Alpha* period**	***Delta* period**	***Omicron* period**
**Overall**	AC-sick-leave	1,204	934	962	1,286	2,109
	CS-sick-leave	40	21	21	29	140
	SARS-CoV-2	593	213	246	396	2,455
**Healthcare**	AC-sick-leave	1,670	1,326	1,328	1,715	2,982
	CS-sick-leave	81	41	42	59	287
	SARS-CoV-2 [a]	185	202	147	158	253
**Education**	AC-sick-leave	1,637	1,081	1,217	1,794	3,398
	CS-sick-leave	18	9	9	15	63
	SARS-CoV-2 [a]	242	223	261	236	268

a Lower completeness of workplace information during the *omicron* period, see supplementary file S2.
