# Supplementary material for: Association between work sick-leave absenteeism and SARS-CoV-2 notifications in the Netherlands during the COVID-19 epidemic
Source: Eur J Public Health. 2024 Mar 21;34(3):497–504. doi: 10.1093/eurpub/ckae051 (PMC11161148; doi:10.1093/eurpub/ckae051)
Supplement: ckae051_Supplementary_Data [file ckae051_supplementary_data.zip › ckae051_Supplementary_Data/ejph-2023-08-om-0463-File010.pdf]

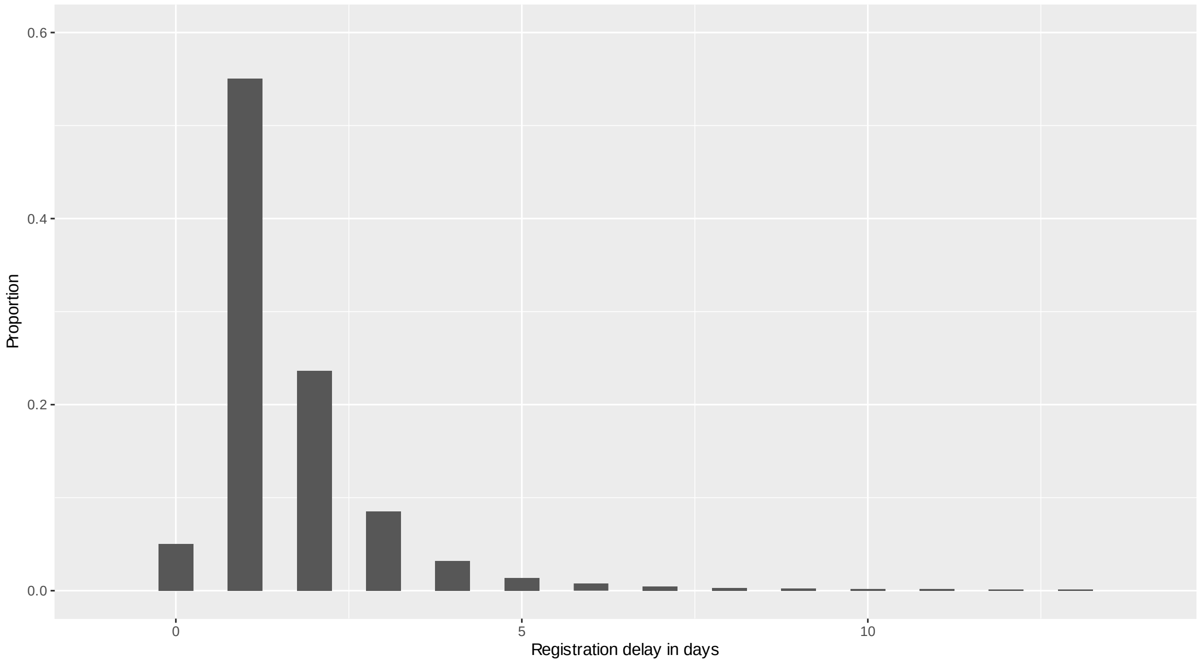


***Supplementary file S7. Retrieval delay SARS-CoV-2 notifications.*** *This figure shows the difference between the positive SARS-CoV-2 test result date and the retrieval date for all notifications during the total study period.*
