# Supplementary material for: Association between work sick-leave absenteeism and SARS-CoV-2 notifications in the Netherlands during the COVID-19 epidemic
Source: Eur J Public Health. 2024 Mar 21;34(3):497–504. doi: 10.1093/eurpub/ckae051 (PMC11161148; doi:10.1093/eurpub/ckae051)
Supplement: ckae051_Supplementary_Data [file ckae051_supplementary_data.zip › ckae051_Supplementary_Data/ejph-2023-08-om-0463-File011.pdf]

Supplementary file S8. Highest Spearman correlation coefficients at optimal lags [a] between the sick-leave and SARS-CoV-2 weekly notification rates during the study period, when using the registration data of sick-leave and publication date of SARS-CoV-2 weekly notification rate.

**Sector**		**Overall**		**Healthcare**		**Education**	
**Type of sick-leave**		All-cause	COVID-19-specific	All-cause	COVID-19-specific	All-cause	COVID-19-specific
**Total study period**	Optimal lag	-1	0	-1	0	-1	0
	Correlation coefficient	0.84, 95% CI [0.77, 0.89]	0.96, 95% CI [0.94, 0.97]	0.74, 95% CI [0.63, 0.82]	0.80, 95% CI [0.72, 0.86]	0.60, 95% CI [0.45, 0.71]	0.70, 95% CI [0.58, 0.79]
**Wildtype period**	Optimal lag	-2	0	-1	-1	0	1
	Correlation coefficient	0.87, 95% CI [0.76, 0.93]	0.90, 95% CI [0.81, 0.95]	0.87, 95% CI [0.76, 0.93]	0.88, 95% CI [0.77, 0.94]	0.84, 95% CI [0.71, 0.92]	0.82, 95% CI [0.68, 0.91]
***Alpha* period**	Optimal lag	-3	0	-3	0	-2	0
	Correlation coefficient	0.63, 95% CI [0.29, 0.83]	0.94, 95% CI [0.85, 0.97]	0.67, 95% CI [0.34, 0.85]	0.84, 95% CI [0.65, 0.93]	0.47, 95% CI [0.00, 0.75]	0.79, 95% CI [0.56, 0.91]
***Delta* period**	Optimal lag	-2	-1	-1	0	-3	0
	Correlation coefficient	0.85, 95% CI [0.68, 0.93]	0.94, 95% CI [0.88, 0.98]	0.86, 95% CI [0.70, 0.93]	0.89, 95% CI [0.76, 0.95]	0.85, 95% CI [0.69, 0.93]	0.86, 95% CI [0.71, 0.94]
***Omicron* period**	Optimal lag	+1	0	0	0	+1	+1
	Correlation coefficient	0.67, 95% CI [0.22, 0.89]	0.93, 95% CI [0.80, 0.98]	0.52, 95% CI [-0.02, 0.82]	0.73, 95% CI [0.33, 0.91]	0.57, 95% CI [0.06, 0.84]	0.67, 95% CI [0.21, 0.88]

[a] Lag in weeks; the optimal lag being the lag with the highest correlation coefficient (negative lags: sick-leave in the weeks preceding SARS-CoV-2 weekly notification rate, positive lags: sick-leave in the weeks after SARS-CoV-2 weekly notification rate).
