# Supplementary figures and images for: Association between work sick-leave absenteeism and SARS-CoV-2 notifications in the Netherlands during the COVID-19 epidemic
Source: Eur J Public Health. 2024 Mar 21;34(3):497–504. doi: 10.1093/eurpub/ckae051 (PMC11161148; doi:10.1093/eurpub/ckae051)

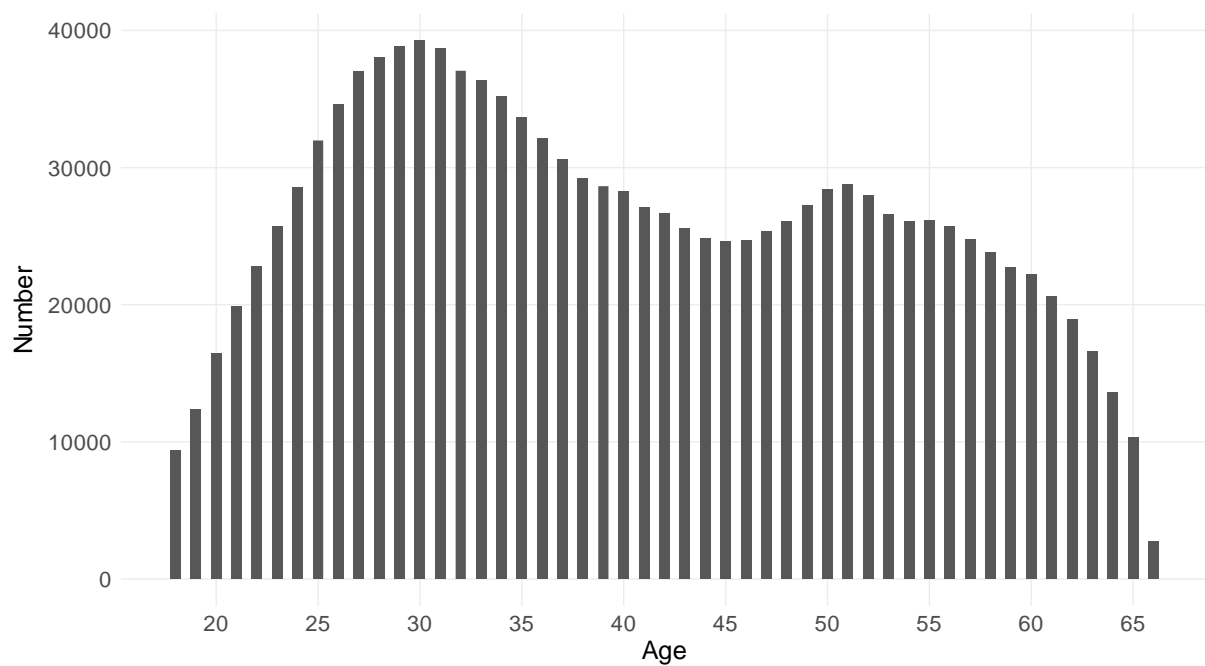

***Supplementary file S4. Age distribution of the sick-leave dataset.***

Supplement: ckae051_Supplementary_Data [file ckae051_supplementary_data.zip › ckae051_Supplementary_Data/ejph-2023-08-om-0463-File007.pdf]

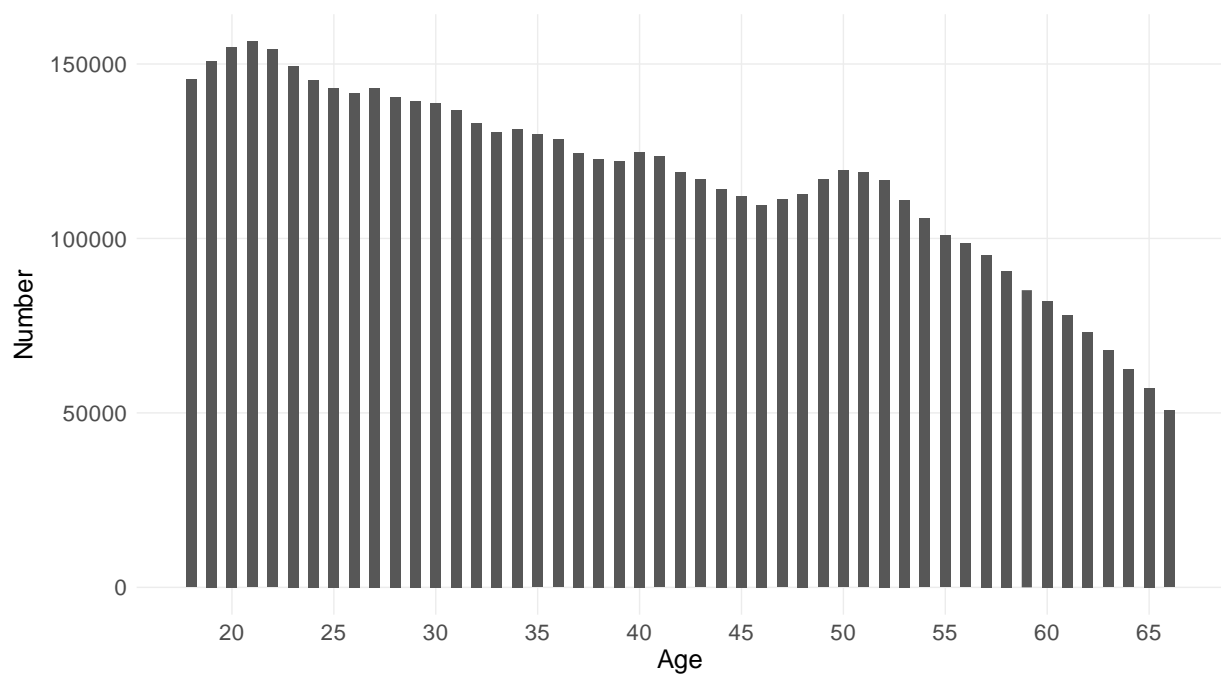

***Supplementary file S5. Age distribution of the SARS-CoV-2 notifications dataset.***

Supplement: ckae051_Supplementary_Data [file ckae051_supplementary_data.zip › ckae051_Supplementary_Data/ejph-2023-08-om-0463-File008.pdf]
